# Cytogenetic characterization of three Balistoidea fish species from the Atlantic with inferences on chromosomal evolution in the families Monacanthidae and Balistidae

**DOI:** 10.3897/compcytogen.v5i1.1141

**Published:** 2011-05-05

**Authors:** Lorena Corina Bezerra de Lima, Pablo Ariel Martinez, Wagner Franco Molina

**Affiliations:** Department of Cell Biology and Genetics, Centro de Biociências, Universidade Federal do io Grande do Norte, Campus Universitário, 59078 – 970 Natal, RN, Brazil

**Keywords:** Balistoidea, fish cytogenetics, karyotype evolution, Tetraodontiformes

## Abstract

The Tetraodontiformes are the most derived group of teleostean fish. Among other apomorphies, they are characterized by a high degree of fusions or significant bone loss in the head and body. In the early phylogenetic proposals presented for this order, the families Balistidae and Monacanthidae have been unanimously considered to be closely related. Although they have moderate species diversity, they are scarcely known in cytogenetic aspect and chromosomal pattern comparisons between these groups have yet to be established. The species *Cantherhines macrocerus* (Hollard,1853), *Cantherhines pullus* (Ranzani, 1842) (Monacanthidae) and *Melichthys niger* (Bloch, 1786) (Balistidae) were cytogenetically analyzed using conventional (Ag-impregnation, C-banding, CMA3- and DAPI-fluorescence) and molecular (FISH with an *18S rDNA* probe) cytogenetic protocols. The karyotypes of all three species were very similar possessing diploid chromosome numbers 2n = 40 and composed exclusively of acrocentric chromosomes. Single NOR-bearing pair as well as positive heterochromatic blocks at pericentromeric regions were identified in the karyotypes of the three species studied. NOR-bearing sites were positively labeled after Ag-impregnation, C-banding, CMA3-fluorescence and FISH with an *18S rDNA* probe but were negative after DAPI-fluorescence. Such remarkable shared conspicuous chromosomal characters corroborate either close phylogenetic relationship of these families, previously established by morphological and molecular data, or rather conservative nature of karyotype differentiation processes. The later hypothesis, however, appears less probable due to centric or *in tandem* fusions documented for another Balistoidea species.

## Introduction

The order Tetraodontiformes, which stands out among marine fish for its marked diversity, is composed of approximately 430 species distributed in nine families (Nelson 2006). This group is the most recent branch of Neoteleostean radiation, representing a post-Perciformes lineage (Elmerot et al. 2002) and although it is generally recognized as a monophyletic group, the relationships between its families and genera have yet to be defined (Holcroft 2004).

Among the Tetraodontiformes, the superfamily Balistoidea (leatherjackets) includes the families Balistidae (triggerfish) and Monacanthidae (filefish), with a fossil record that dates back to the Early Eocene and probably to the Late Cretaceous (Frickhinger 1995, Santini and Tyler 2003). Based on morphological similarities (e.g., osteological and myological characters), molecular studies using RAG1 gene sequences and DNA content data (Winterbottom 1974, Brainerd et al. 2001, Holcroft 2004, 2005), and more recently analysis of complete mitochondrial genomes (mitogenome) (Yamanoue et al. 2008), members of families Balistidae and Monacanthidae are considered monophyletic sister groups.

To date nearly 60 species of Tetraodontiformes have been cytogenetically studied (Sá-Gabriel and Molina 2005). Cytogenetic analyses have been carried out in 15 Balistidae species, most from the Pacific region, and only few from the Western Atlantic. A total of ten Monacanthidae species are karyotyped, being one from the Brazilian coast.

In this work, we revise cytogenetic data for *Melichthys niger* (Bloch, 1786) (Balistidae) and describe the karyotype and other chromosomal characteristics for *Cantherhines macrocerus* (Hollard, 1853) and *Cantherhines pullus* (Ranzani, 1842) (Monacanthidae), to compare the chromosomal patterns of the families Balistidae and Monacanthidae, estimating their divergence level.

## Material and methods

We analyzed 42 specimens of *Melichthys niger* and 14 of *Cantherhines macrocerus*, collected in the Saint Peter and Saint Paul’s Archipelago (00º55’15” N, 029º20’60” W), 1010 km from the Brazilian northeastern coast and about 1.824 km) from the African coast, and two specimens of *Cantherhines pullus* collected in the coastal region of Salvador (12º58’S, 38º31’W), Bahia state, northeastern Brazil (Fig. 1).

**Figure:**
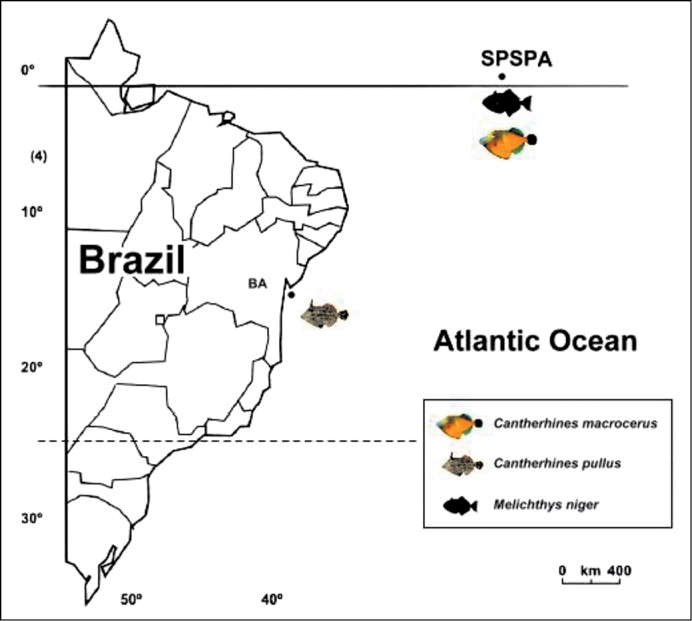
**Figure 1.** Map showing the geographic collection points of the species *Melichthys niger*, *Cantherhines macrocerus* and *Cantherhines pullus*. SPSPA – Saint Peter and Saint Paul’s Archipelago; BA – Bahia state.

The specimens were subjected to mitotic stimulation as proposed by Molina (2001) for a period of 24-48 hours. Mitotic chromosomes were obtained following the protocol developed by Gold et al. (1990). Cell suspensions obtained from fragments of the anterior portion of specimens’ kidney were spread onto slides with a film of water heated at 60ºC. The active nucleolus organizer regions (NOR) were identified by silver nitrate staining as described by Howell and Black (1980), while the heterochromatic regions were visualized by C-banding (Sumner 1972). The FISH technique (according to Pinkel et al. 1986) was performed using an *18S rDNA* probe from *Prochilodus argenteus*, Agassiz, 1829 (Hatanaka and Galetti 2004), labeled with biotin-14-dATP by nick translation according to the manufacturer’s instructions (BioNickLabeling System; Invitrogen, Carlsbad, CA, U.S.A.). The hybridization signal was detected by the streptavidin-fluorescein isothiocyanate conjugate. Sequential staining with AT- specific 4’-6-diamidino-2-phenylindole (DAPI) and GC-specific Chromomycin A3 (CMA3) fluorochromes was performed as described by Schweizer (1980). The metaphases were photographed by a DP70 digital image capture system coupled to an Olympus BX50 epifluorescence microscope. About thirty metaphases of each specimen were analyzed to determine the modal number of mitotic chromosomes and the karyotype. The chromosomes were classified according to centromere position and organized into decreasing size order, as proposed by Levan et al. (1964).

## Results

The specimens of *Melichthys niger* showed 2n=40 chromosomes and a karyotype consisting of 20 pairs of acrocentric (a) chromosomes (NF=40) (Fig. 2a). The presence of a conspicuous secondary constriction was observed in the interstitial position on the long arm of the chromosome pair No. 2, corresponding to the nucleolus organizer regions (NORs), identified by Ag-NOR sites and by *in situ* hybridization with an *18S rDNA *ribosomal probes (Fig. 2, upper boxes). The heterochromatic blocks were reduced in size and dispersed in the pericentromeric regions in most of the chromosome pairs (Fig. 2b). The NORs were heterochromatic and CMA3 positive and DAPI negative (Fig. 2, upper boxes).

**Figure:**
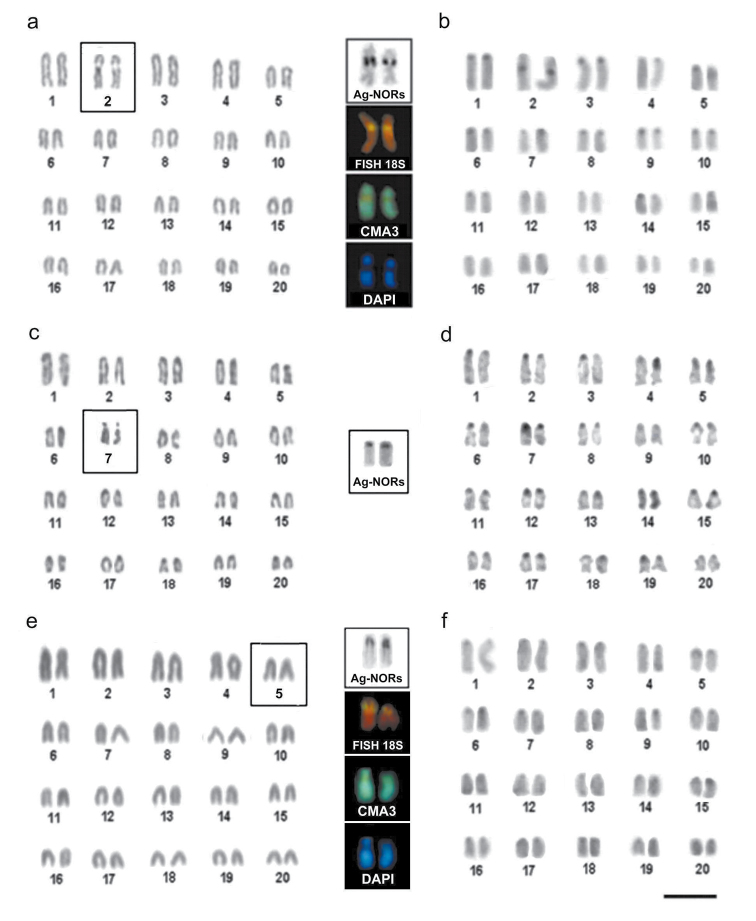
**Figure 2.** Karyotypes of *Melichthys niger* (**a, b**)*,* *Cantherhines macrocerus* (**c, d**) and *Cantherhines pullus* (**e, f**), arranged from Giemsa stained (**a, c, e**) and C-banded chromosomes (**b, d, f**). In the center highlighted are the NOR-bearing pairs of analyzed species (2nd, 7th and 5th, respectively) after Ag-NOR staining, *in situ* hybridization with an *18S rDNA* probe, CMA3 and DAPI fluorescence. Bar = 5µm.

The specimens of *Cantherhines macrocerus* (Monacanthidae) had 2n=40 chromosomes and karyotype composed of a acrocentric chromosomes (Fig. 2c). Ag-NOR sites were located in the pair No. 7 in interstitial region, near the centromere (Figure 2, middle box). C-banding revealed heterochromatic blocks distributed in the pericentromeric region in most of the chromosome pairs (Figure 2d) and more intensively stained on the secondary constriction of the NOR-bearing pair. In this species, experiments using FISH probes and fluorochrome staining were unsuccessful.

The specimens of *Cantherhines pullus* showed 2n=40 chromosomes and karyotype composed entirely of acrocentric chromosomes (Fig. 2e). The NORs were identified at the pericentromeric position of pair No. 5, as revealed by Ag-NOR-staining and FISH with an *18S rDNA *probes (Fig. 2, lower box). The heterochromatic regions were distributed in centrometric and pericentromeric positions in most of the chromosomes. The NORs sites were heterochromatic (Fig. 2f), and CMA positive and DAPI negative (Fig. 2, lower box). None of the karyotypes displayed sex-related chromosome heteromorphism.

## Discussion

Six out of the ten karyotyped species in the family Monacanthidae have diploid numbers ranging from 2n = 33/34 to 36 chromosomes. Such low 2n numbers have been a noticeable characteristic for Monacanthidae species. The present data for *Cantherhines macrocerus* and *Cantherhines pullus* increase the range of the highest 2n for representatives of this family. Surveys involving a larger number of genera may confirm a possible basal karyotype with 40 chromosomes for this family, showing on average lower diploid values than those of the Balistidae. Based on chromosomal number, *Cantherhines macrocerus* and *Cantherhines pullus* would be placed in the family Balistidae.

The karyotype of the individuals of *Cantherhines macrocerus* from the Saint Peter and Saint Paul’s Archipelago was similar to those described for specimens from the coast of Rio de Janeiro (Pauls 1993), even though these populations were 2000 km apart. It is not clear whether the common karyotype found in *Cantherhines macrocerus* populations is maintained due to gene flow by the transport of their pelagic larvae by ocean currents, as has been commonly identified for a number of reef species (Rocha 2003, Feitoza et al. 2005), or if they were isolated too recently to accumulate observable chromosomal differences.

In this study, the data obtained for *Melichthys niger* corroborate results described for the species earlier (Sá-Gabriel and Molina 2005). However, the use of a larger number of individuals of this species, observation of lower chromatid condensation and the use of complementary cytogenetic protocols, such as CMA3/DAPI fluorochromes and FISH with an *18S rDNA* probes, allow more precise localization of the NOR-bearing pair to the 2nd largest pair of the karyotype.

The chromosomal characteristics observed in *Cantherhines macrocerus,* *Cantherhines pullus* and *Melichthys niger*, like the presence of single NOR and the pericentromeric heterochromatin blocks as reported in other Tetraodontiformes (Grützner et al. 1999, Mandrioli 2000, Fischer et al. 2000), Perciformes (Caputo et al. 2001, Molina 2007), Mugiliformes (Nirchio et al. 2009), Beryciformes (Bacurau and Molina 2004), corroborate the hypothesis that these are ancestral characteristics for each of these clades and therefore not exclusive to the families Monacanthidae and Balistidae.

In the karyotypes of species under our study, as well as in other representatives of Balistidae and Monacanthidae (Sá-Gabriel and Molina 2004), extra-pericentromeric heterochromatic regions were present only when adjacent to or associated with the major ribosomal sites. Given that the constitutive heterochromatin blocks in fish chromosomes are often associated with karyotype diversification (Mantovani et al. 2000, Molina and Bacurau 2006), it is possible that these regions present preferentially in peri-centromeric position in chromosomes of Balistoidea species and they are involved in the karyotypic differentiation of this group. The presence of heterochromatin associated with NORs in adjacent or interspersed regions (Pendás et al. 1993), as shown in the NOR-bearing pair in *Melichthys niger*, may contribute to the occurrence of structural rearrangements involving NOR-bearing pairs (Vicari et al. 2003). Indeed, chromosome fusions have been commonly identified as the main mechanism of the karyotype diversification within this clade (Kitayama and Ojima 1984). Although a physical mapping of telomeric sequences is not yet available, the lower 2n in both Balistidae and Monacanthidae corroborate that centric and/or *in* *tandem* fusion events, followed by pericentric inversions, seem to have been important mechanisms in the karyotype differentiation of these post-Perciformes group.

Repetitive sequences and transposition elements are closely related to the heterochromatic regions, and although account for less than 10% of the genome of the Tetraodontiformes studied (Brenner et al. 1993), they may be effective promoters of chromosomal breaks, deletions, inversions and amplifications (Lim and Simmons 1994, Fischer et al. 2000). Knowledge regarding aspects of the Balistoidea genome is still limited, although it is known that, like Tetraodontidae, this clade is composed of species with compact genomes that evolved independently (Brainerd et al. 2001). The Tetraodontidae genome has been extensively studied (Crolius et al. 2000), revealing that the small amount of repetitive sequences is located in the centromeres and arms of a chromosomes. This physical disposition seems to be present in the chromosomes of Balistoidea and could help to explain why centromere and telomere regions would be prone to ocurrence of *in tandem* or centric fusions rearrangements. In addition to the aforementioned data, a large number of acrocentric chromosomes and FN near or slightly higher than the diploid chromosome values were observed. These characteristics are not common in other families of the same order, such as the Tetraodontidae (Sá-Gabriel and Molina 2005), where pericentric inversions seem to have occurred more frequently.

Among the Perciformes, the presence of single interstitial NORs is common, representing an ancestral condition (Affonso et al. 2001). Similarly, the presence of heterochromatin restricted to the centromeric regions is also a typical cytogenetic trait of this group (Molina and Galetti 2002). The location and frequency of NORs sites and heterochromatic regions in *Cantherhines macrocerus*, *Cantherhines pullus* and *Melichthys niger* are consistent with the general pattern found in Perciformes species, demonstrating the apparent conservativeness of these characters.

The set of cytogenetic characters already available in Monacanthidae and Balistidae species indicate a greater karyotypic similarities and common tendencies of karyotype evolution with other groups of the order Tetraodontiformes, corroborating previous analyses based on morphological and molecular data (Winterbottom 1974, Brainerd et al. 2001, Holcroft 2005, Yamanoue et al. 2008).
